# Application of Glycerine in Variola

**Published:** 1859-05

**Authors:** T. A. Demme


*Application of Glycerine in Variola.* From the German, by T. A. DEMME, M. D.

The terrible disfigurement that Variola often inflicts upon its victims, is one cause of the dread and horror that man instinctively feels at the approach of this loathsome blight. Notwithstanding the earnest endeavors of the physician to discover some therapeutic application that may protect his patient against the pitting and seaming which this disease so often leaves, we are compelled to admit, that comparatively little has been done towards accomplishing this object—mercurial plaster and ointment and penciling with astringent solutions failing to insure against deformity.

Dr. Posner, editor of the *Medic. Central Zeit.*, in the January number, 1859, of his journal, recommends the application of Glycerine in Variola, affirming that it protects and secures the patient against the variola deformity.

He was led to the use of glycerine, in consequence of the entreaties of his patients for some application that would relieve the distressing pain in the pustules; for this purpose he directed the annointing of the painful parts, every two hours, with pure glycerine; his anticipations were answered—the pain and tension being overcome.

It chanced that the first two patients, upon whom the remedy was tested, were completely covered with pustules, which, upon the face, were confluent. Great deformity was expected—but when the scabs fell off, contrary to every anticipation, the scars that remained were small and on a level with the skin—they were, however, of such a dark color that the patients looked like mulattoes.

Out of instinct, not in obedience to direction, the convalescents continued the application of the glycerine, and after six weeks, the discoloration had disappeared, and the scars were scarcely visible.

Since then a number of patients have been thus protected.

Great care must be taken that the glycerine is perfectly pure.—*Medical & Surgical Reporter.*

